# Numerical Simulation Study on the Temperature Rise Characteristics of Asphalt Pavement During Hot In-Place Recycling

**DOI:** 10.3390/ma19102096

**Published:** 2026-05-16

**Authors:** Chuanyi Ma, Jizhe Zhang, Bokai Liu, Yuechao Zhao, Peng Zhou, Liting Yan, Zhanyong Yao, Weidong Cao

**Affiliations:** 1 Shandong Hi-Speed Group Co., Ltd., Jinan 250098, China; 2 School of Qilu Transportation, Shandong University, Jinan 250002, China; 3 Shandong Provincial Communications Planning and Design Institute Group Co., Ltd., Jinan 250101, China; 4 School of Materials Science and Engineering, University of Jinan, Jinan 250022, China; 5 Shandong Hi-Speed Dongying Development Co., Ltd., Dongying 257092, China

**Keywords:** asphalt pavement, hot in-place recycling, heating parameters, heat penetration efficiency, numerical simulation

## Abstract

The heating efficiency of hot in-place recycling for asphalt pavements directly depends on parameters such as hot air temperature, hot air velocity, and heating machinery travel speed. However, the interactions among these parameters and their influence mechanism on deep-layer temperature remain unclear. In this study, a three-dimensional transient heat transfer model of an asphalt pavement was established to simulate the effects of different parameters under intermittent heating conditions, including hot air temperature, hot air velocity, and travel speed, on the pavement surface temperature and temperature at a depth of 4 cm. The results indicate that travel speed exhibits the most sensitive effect on temperature, with an absolute regression coefficient of 8.5 °C·min/m. A reduction of 0.5 m/min in travel speed increases the deep-layer temperature by approximately 4.25 °C. Every 50 °C increase in hot air temperature raises the deep-layer temperature by about 5.75 °C. The effect of hot air velocity on temperature is relatively small within the range of 12 to 14 m/s. The heat penetration efficiency increases monotonically as the hot air temperature and velocity increase or the travel speed decreases. This study reveals the quantitative relationship between heating parameters and temperature responses, providing a theoretical basis for parameter optimization in hot in-place recycling processes.

## 1. Introduction

During long-term service, asphalt pavement is susceptible to structural and functional distress, including cracking, rutting, and raveling on the surface layer, due to the coupled effects of traffic loading and environmental factors, such as temperature cycling, ultraviolet aging, and moisture damage [1,2]. Although traditional milling and overlay techniques can completely remove surface distress, they consume substantial amounts of new aggregates and asphalt binder, generate large quantities of reclaimed asphalt pavement (RAP) materials, and are associated with high carbon emissions, significant energy consumption, and prolonged traffic disruption [3,4]. In this context, hot in-place recycling (HIR) has emerged as a critical technology for the preventive maintenance and functional rehabilitation of asphalt pavement, owing to its distinct advantages, including 100% reuse of existing asphalt mixture, substantial reduction in new material consumption, lower environmental impact, and shortened construction time [5]. The hot in-place recycling process involves in situ heating and softening of the existing pavement using heating units, followed by scarifying, the addition of rejuvenators and new mixture, mixing, paving, and compaction to form a new surface layer [6]. With the advancement of China’s “dual carbon” strategy and the widespread adoption of green maintenance concepts, the application of hot in-place recycling technology in asphalt pavement maintenance has been expanding, accompanied by continuous improvements in equipment and construction processes, particularly on expressways and arterial highways [7].

The quality control of HIR construction largely depends on the effectiveness of the heating process in regulating the pavement temperature field [8]. The adequacy and uniformity of heating directly determine the ease of scarifying, the degree of blending between old and new materials, and the quality of interlayer thermal bonding [9,10]. Specifically, insufficient heating fails to adequately soften the aged asphalt mixture, leading to difficulties during scarifying, uneven material distribution, and formation of weak bonding interfaces between the recycled layer and the existing pavement, thereby compromising structural integrity [11]. Conversely, excessive heating temperatures or prolonged heating durations may accelerate asphalt aging, increasing the volatilization of light components and even heat-induced damage, such as burning or oil bleeding, resulting in irreversible deterioration of material properties [12]. Therefore, accurately characterizing the temperature rise behavior of asphalt pavement under moving heat sources and systematically elucidating the quantitative relationships between heating process parameters and pavement thermal responses are fundamental to achieving precise heating control, optimizing construction parameters, and ensuring the quality of the recycled layer [13]. Notably, the temperature at a depth of 4 cm, which typically corresponds to the critical influence zone of the recycled layer, directly affects the scarifying efficiency and interlayer thermal bonding quality, serving as a core indicator for heating quality control [14]. Without a comprehensive understanding of temperature rise characteristics, it becomes quite challenging to formulate a scientific heating strategy, thereby compromising the long-term performance and durability of recycled pavements [15].

Extensive research has been dedicated to heat transfer mechanisms in HIR of asphalt pavements from both experimental and numerical simulation perspectives [16,17]. Experimental studies have employed full-scale field tests and laboratory heating simulations using thermocouples and infrared thermography to measure the dynamic temperature fields on the pavement surface and within the layer under various heating conditions [18]. These studies have analyzed the effects of factors such as heating power, heating duration, ambient temperature, and wind speed on heating rates, temperature gradients, and peak temperatures. In parallel, numerical simulations have advanced significantly with the development of finite element methods, computational heat transfer, and multiphysics coupling techniques [19]. Researchers have established unsteady-state heat transfer models for asphalt pavements that incorporate moving heat sources, temperature-dependent nonlinear variations in material thermal properties, and complex boundary conditions, including surface convection and solar radiation. These models have been employed to simulate temperature field evolution under different heating methods, such as hot-air circulation, infrared heating, and microwave heating [19]. Some studies have further explored the influence of macro-level parameters on heating performance, including ambient temperature, initial pavement temperature, and overall heating unit power [20]. However, existing research has primarily focused on macroscopic parameters like total heating power or heating duration, while systematic investigations into the relationships between finely controllable process parameters and the pavement temperature field remain limited, particularly heating vehicle speed, hot air temperature, and hot air velocity. In practice, the hot air temperature and velocity collectively determine the convective heat transfer intensity between hot air and the pavement surface, whereas the heating vehicle speed governs the duration of heat application and the accumulation of thermal energy [21]. These three parameters are mutually coupled and jointly influence the temperature rise process. There is currently a lack of systematic numerical simulations and parametric sensitivity analyses addressing these three parameters, making it difficult to identify their relative importance and optimal ranges.

In view of the above, this study aims to develop a three-dimensional unsteady-state heat transfer model for the heating process in HIR of asphalt pavement based on heat transfer theory and the finite element method. The model will account for the temperature-dependent nonlinear variations in the thermal properties of the asphalt mixture, dynamic loading of moving heat source boundary conditions, and surface convective and radiative heat transfer to realistically represent the thermal process in actual construction. The study will focus on the effects of three key process parameters on the pavement temperature field, including hot air temperature, hot air velocity, and heating vehicle speed. The pavement surface temperature and the temperature at a depth of 4 cm will be selected as the primary evaluation indicators. The 4 cm depth corresponds to the critical influence zone of the recycled layer, where the temperature level directly affects scarifying efficiency and interlayer thermal bonding quality. Through single-factor analysis, the response characteristics of heating rate, time required to reach target temperatures, and temperature gradient within the layer will be examined under variations in each parameter. Through multi-factor comparison and parametric sensitivity analysis, the influence of each parameter on the temperature field will be quantitatively assessed, and the relative importance and reasonable ranges of these parameters will be identified. Simulation conditions will be defined based on typical equipment parameters and practical engineering conditions to ensure the applicability of the research findings.

The outcomes of this study are expected to provide theoretical foundations and data support for the optimization of heating process parameters in HIR construction. By clarifying the appropriate matching relationships between heating vehicle speed, outlet air temperature, and outlet air velocity, the findings will facilitate the achievement of satisfactory heating quality while reducing energy consumption and minimizing the risk of thermal damage. Specifically, ensuring that the surface and 4 cm depth temperatures meet construction requirements with a uniform temperature distribution. This will contribute to the advancement of HIR technology toward more precise and intelligent construction practices. Furthermore, the numerical simulation methodology and analytical framework developed in this study are highly extensible and can serve as references for temperature field analyses of other moving heat source heating processes, such as infrared heating and microwave heating, thereby supporting the enhancement of green maintenance technologies for asphalt pavements.

## 2. Finite Element Model Establishment

### 2.1. Asphalt Pavement Material Parameters

**Figure 1:**
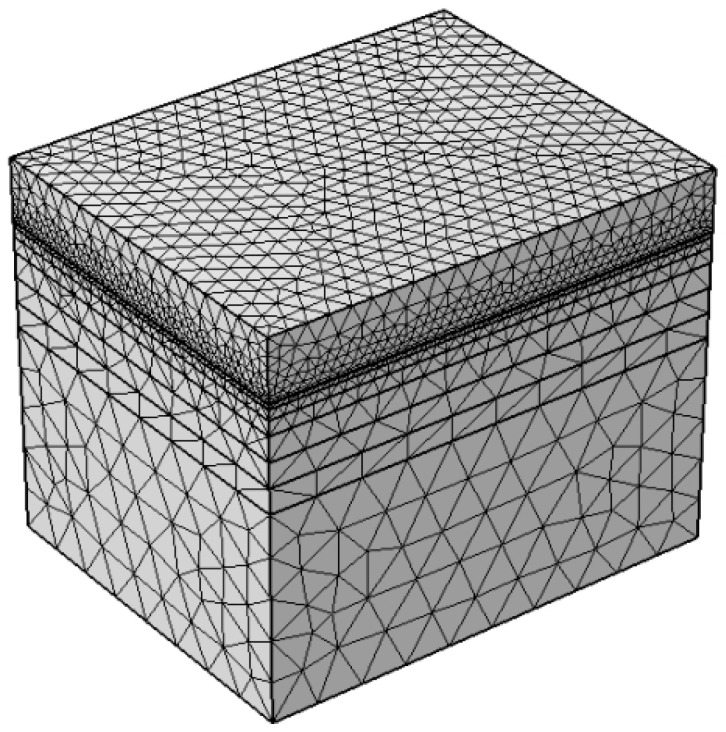
Finite element model of asphalt pavement.

In this study, the selected pavement structure and thermal physical parameters are as follows: 4 cm SMA-13 modified asphalt concrete (surface course), 6 cm AC-20 (middle course), 8 cm AC-25 (bottom course), 36 cm 5% cement-stabilized macadam (base), 18 cm graded gravel (subbase), and subgrade soil. The thermophysical parameters for the asphalt layers were taken as density 2560 kg/m^3^, specific heat capacity 1000 J/(kg·K), and thermal conductivity 1.2 W/(m·K). These were as follows: for the cement-stabilized base, 2200 kg/m^3^, 900 J/(kg·K), and 1.5 W/(m·K); and for the subgrade soil, 1800 kg/m^3^, 1200 J/(kg·K), and 1.0 W/(m·K) [22]. Based on the pavement structure type and the thermal physical parameters, a finite element analysis geometric model of 300 mm × 300 mm × 500 mm was established using COMSOL Multiphysics finite element software (COMSOL6.4), as shown in Figure 1.

### 2.2. Simulation Conditions

The temperature field variation in pavement structures is governed by two main factors: the material’s thermal properties and initial temperature, including external conditions such as ambient temperature, hot air temperature, and velocity from the heating unit; and the unit’s travel speed. To investigate the effects of different influencing factors on the temperature field of asphalt pavement, various values were selected for each influencing parameter, based on typical operating capabilities of hot in-place recycling equipment and common engineering practice on expressways. The parameter selection and configurations are presented in Table 1. The base values are 500 °C, 13 m/s, and 3.0 m/min. In the single-factor analyses, when one parameter is varied, the other two are held at these base values. In terms of setting the boundary conditions, the top surface considers heat exchange with the external environment, including solar radiation, convective heat transfer with ambient air, and longwave radiation. For the bottom boundary, a constant temperature boundary condition is applied to represent the thermal influence of the deep subgrade, assuming that the temperature variation at depth is minimal and essentially stable over the simulation period. Since the lateral dimensions of the model are much larger than the heat penetration depth, adiabatic boundary conditions are applied to the side boundaries.

**Figure 2:**
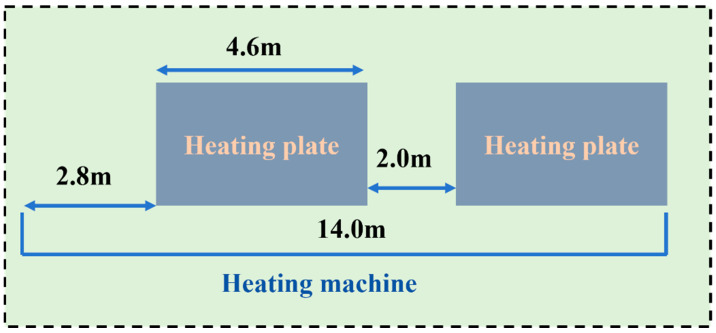
Schematic diagram of the heating machine structure.

In actual field construction, three heating machines are used, with a spacing of 1–2 m between adjacent machines. In the numerical simulation of this study, the spacing between heating machines is set to 1.5 m. The total length of a single heating machine is 14 m, and its specific structure is shown in Figure 2.

**Figure 3:**
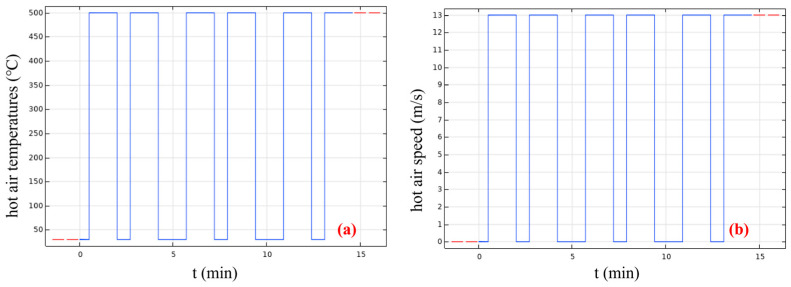
The relationship between heating time and hot air temperature (**a**)/velocity (**b**).

Based on this, the time required for the heating machine to pass over the pavement at different vehicle speeds can be calculated. In the numerical simulation, different heating conditions are realized by setting the temperature and velocity of the hot air in different time periods. For this purpose, a 300 mm × 300 mm × 100 mm air domain was established to apply heat for subsequent simulation of hot air. The air temperature was input through fixed boundary conditions, that is, a constant temperature value was directly applied at the specified boundaries. Here, the air is not a moving hot air mass, but a stable heat flow field formed by boundary temperature control. When the heating box passes over the pavement, the hot air temperature is set to the specific process parameter; when other structures of the heating machine pass over the pavement, the hot air temperature is set to the ambient air temperature. In this study, the summer ambient air temperature is taken as 20 °C. Based on the above settings, the influence curves of heating time on hot air temperature and hot air velocity can be further derived, as shown in Figure 3.

## 3. Results and Discussion

### 3.1. Effect of Heating Parameters on the Pavement Surface Temperature

The surface temperature of the road exhibits a “heating-cooling” sawtooth fluctuation under intermittent heating conditions. The peak value determines the risk of asphalt coking, while the valley value reflects the residual temperature of the surface layer after the heat has been transferred downward. This section will analyze the individual effects of the hot air temperature, the hot air speed, and the movement speed of the heating plate on the pavement surface temperature.

#### 3.1.1. Hot Air Temperature

**Figure 4:**
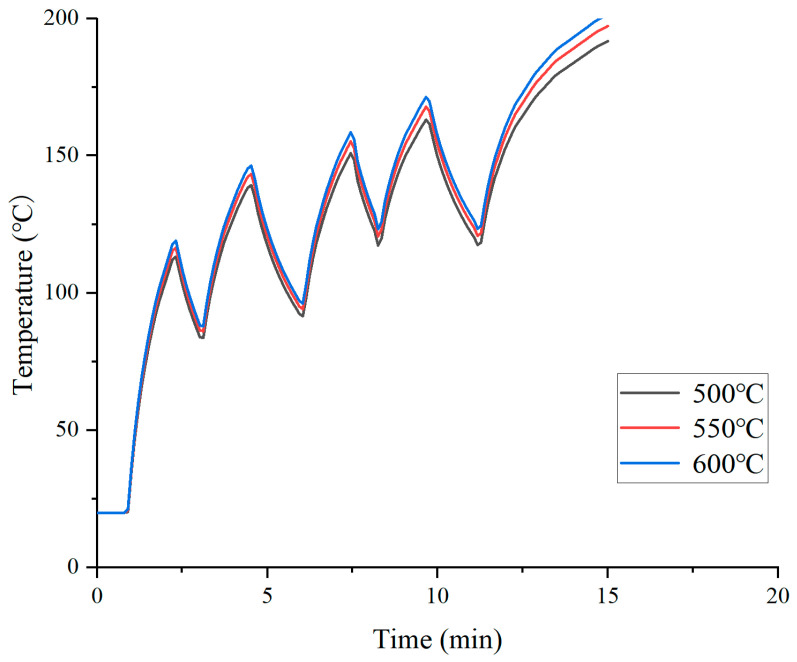
Effect of hot air temperature on pavement surface temperature.

As shown in Figure 4, after heating with hot air temperatures of 500 °C, 550 °C, and 600 °C for 15 min, the final surface temperatures of the road surface reached 191.7 °C, 197.2 °C, and 201.4 °C, respectively. The corresponding temperature increments are approximately 5.5 °C and 4.2 °C, showing a gradually decreasing trend. During each heating cycle of intermittent heating, the surface temperature rise rate under the 600 °C condition is significantly higher than that under the 500 °C condition, but the cooling slopes of the three conditions are similar, indicating that the cooling process is primarily governed by ambient conditions and thermal diffusion in the underlying asphalt layer, independent of the hot air temperature. Notably, under the 600 °C condition, the pavement surface temperature exceeds 200 °C at the end of the heating period, reaching the coking threshold of asphalt. In contrast, the final temperature under the 500 °C condition remains below 200 °C, and the heat penetration depth is limited. Therefore, 550 °C can be taken as a reference value for the safe upper limit, which ensures a relatively high heat flux density while providing a buffer margin for the peak temperature during intermittent heating.

#### 3.1.2. Hot Air Velocity

**Figure 5:**
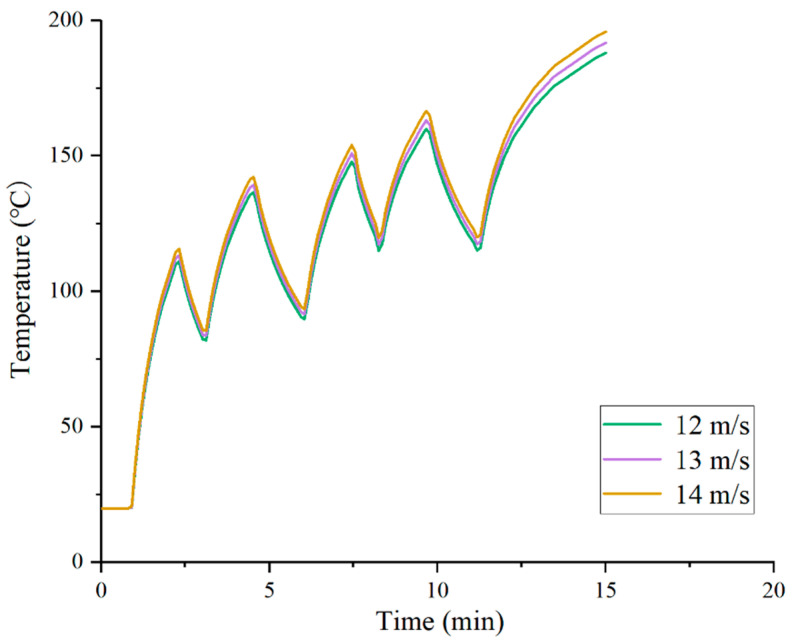
Effect of hot air velocity on pavement surface temperature.

As shown in Figure 5, when the hot air velocity increases from 12 m/s to 14 m/s, the final pavement surface temperature after 15 min of heating rises from 187.9 °C to 195.8 °C, with an increment of approximately 7.9 °C. During the initial heating stage, the surface temperature rise rate under the 14 m/s condition is the highest, but the subsequent amplitude of sawtooth fluctuation is also the largest, indicating that a higher air velocity increases the convective heat transfer coefficient between the hot air and the pavement surface, leading to a substantial accumulation of heat in the surface layer within a short period. However, during the cooling phase of intermittent heating, the surface cooling rate under the 14 m/s condition is also slightly faster than that under the 12 m/s condition, which may be attributed to the larger temperature difference between the hot surface layer and the ambient environment.

Under the 12 m/s condition, the pavement surface temperature remains relatively stable throughout the process, with the smallest sawtooth amplitude, but the overall heating efficiency is low, reaching only 187.9 °C after 15 min, which may fail to meet the requirements of certain recycling processes. The 13 m/s condition exhibits a good balance: a moderate heating rate and a peak-to-trough temperature difference controlled within approximately 25–30 °C, avoiding both the insufficient heating observed at 12 m/s and the overheating risk at 14 m/s. It is recommended to set the hot air velocity in the range of 12.5–13.5 m/s, with fine-tuning based on ambient temperature—appropriately increasing the air velocity under low-temperature conditions to compensate for heat loss.

#### 3.1.3. Heating Vehicle Speed

**Figure 6:**
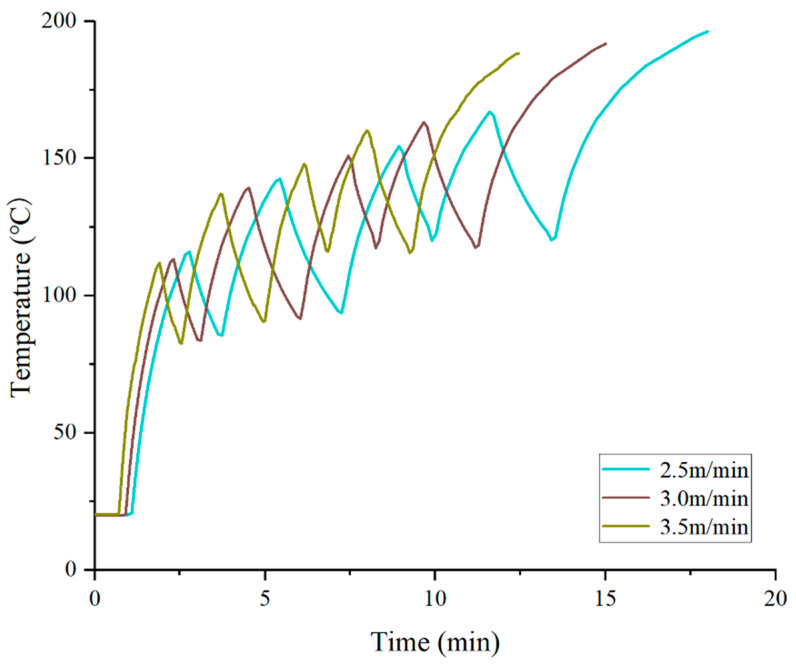
Effect of vehicle speed on pavement surface temperature.

Vehicle speed is the only parameter among the three that is directly related to heating time. As shown in Figure 6, the final pavement surface temperatures corresponding to speeds of 2.5 m/min, 3.0 m/min, and 3.5 m/min are 196.2 °C, 191.7 °C, and 187.7 °C, respectively. For every increase of 0.5 m/min in vehicle speed, the final temperature decreases by approximately 4–5 °C. This indicates that under intermittent heating mode, vehicle speed primarily determines the total heat input during each heating cycle. At low speed, the sawtooth peaks of the pavement surface temperature are significantly higher, and the trough temperatures are also elevated accordingly, because each heating–cooling cycle lasts longer, allowing more time for heat to transfer downward before being replenished by the next heating period.

### 3.2. Effect of Heating Parameters on Deep-Layer Temperature of Pavement

The temperature at a depth of 4 cm reflects the penetration ability of heat into the asphalt layer and is a key indicator for determining whether the regeneration depth meets the requirements. Under intermittent heating conditions, the temperature curve at this point is a smooth ascending type, demonstrating the lag and filtering effect of heat conduction. This section will analyze the individual effects of the hot air temperature, hot air speed, and movement speed of the heating plate on the temperature at a depth of 4 cm.

#### 3.2.1. Hot Air Temperature

**Figure 7:**
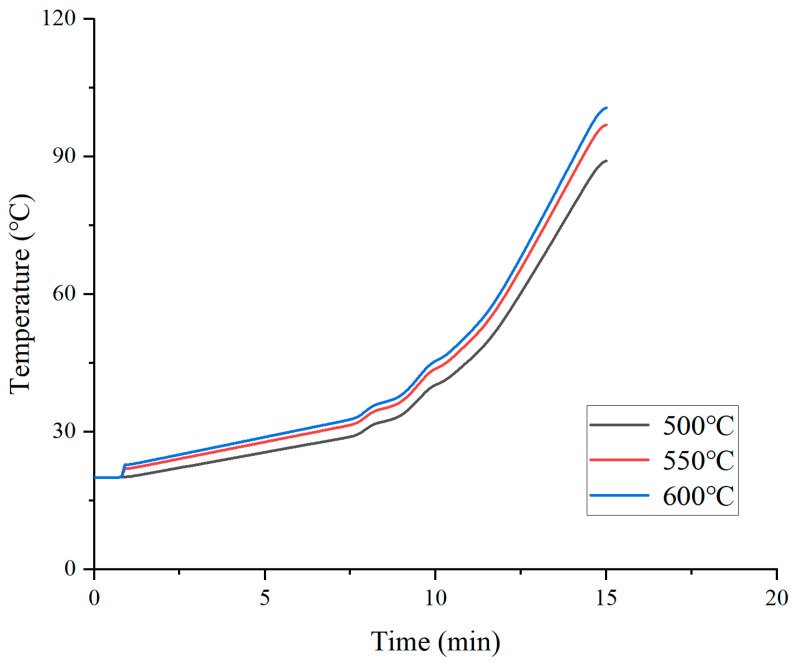
Effect of hot air temperature on the deep-layer temperature of pavement.

The data in Figure 7 show the effect of hot air temperature on the temperature at 4 cm depth. Under the conditions of 500 °C, 550 °C, and 600 °C, the temperatures at a 4 cm depth after 15 min of heating are 89.1 °C, 96.9 °C, and 100.6 °C, respectively. For every 50 °C increase in hot air temperature, the temperature rise at a 4 cm depth is approximately 7.8 °C and 3.7 °C, with the increment decreasing as the temperature rises. This phenomenon can be explained by Fourier’s law of heat conduction: the heat flux density is proportional to the temperature difference, but when the surface temperature becomes excessively high, part of the heat is dissipated to the environment through convection and radiation rather than being transferred entirely into the deeper layers.

Notably, under the 600 °C condition, the temperature at a 4 cm depth is only 3.7 °C higher than that at 550 °C, whereas the surface temperature is 4.2 °C higher. This means that once the hot air temperature exceeds 550 °C, further yield increases significantly, diminishing the marginal benefits for deep-layer heating. In terms of thermal penetration efficiency (i.e., the ratio of the temperature rise at a 4 cm depth to that at the pavement surface), the efficiencies at 500 °C, 550 °C, and 600 °C are 0.46, 0.49, and 0.50, respectively. Therefore, if the goal is to heat the 4 cm depth to above 100 °C, 550 °C is sufficient under the present simulation conditions (hot air velocity 13 m/s, vehicle speed 3.0 m/min). When other parameters are changed, the required temperature should be re-evaluated accordingly. If a temperature of 110 °C is required, the hot air temperature must be increased above 600 °C but with simultaneous enhancement of surface protection measures, such as reducing the hot air velocity or shortening the duration of each heating cycle.

#### 3.2.2. Hot Air Velocity

**Figure 8:**
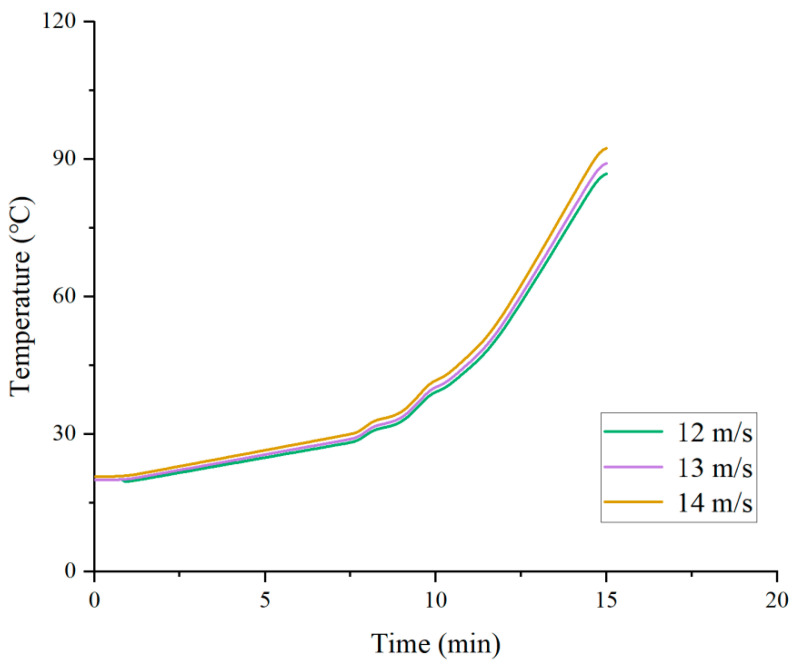
Effect of hot air velocity on the deep-layer temperature of pavement.

The effect of hot air velocity on the temperature at a depth of 4 cm exhibits a monotonically increasing characteristic, as shown in Figure 8. At hot air velocities of 12 m/s, 13 m/s, and 14 m/s, the corresponding final temperatures after 15 min of heating are 86.8 °C, 89.1 °C, and 92.4 °C, respectively. Throughout the entire heating process, the temperature at a 4 cm depth under the 14 m/s condition remains consistently higher than that under the 13 m/s condition, and the 13 m/s condition remains consistently higher than the 12 m/s condition, with no crossover phenomenon. This indicates that within the range of 12 to 14 m/s, increasing the hot air velocity continuously enhances the convective heat transfer coefficient, allowing more heat to be transferred to the asphalt pavement and thereby effectively increasing the deep-layer temperature. Notably, although a higher air velocity leads to faster surface heating, no significant lag in downward heat conduction is observed. The deep-layer temperature shows an increasing trend with higher air velocity from the early stage of heating, indicating that within this velocity range, thermal penetration capacity is positively correlated with air velocity, and the negative effect of “heat retention in the surface layer” does not occur.

#### 3.2.3. Heating Vehicle Speed

**Figure 9:**
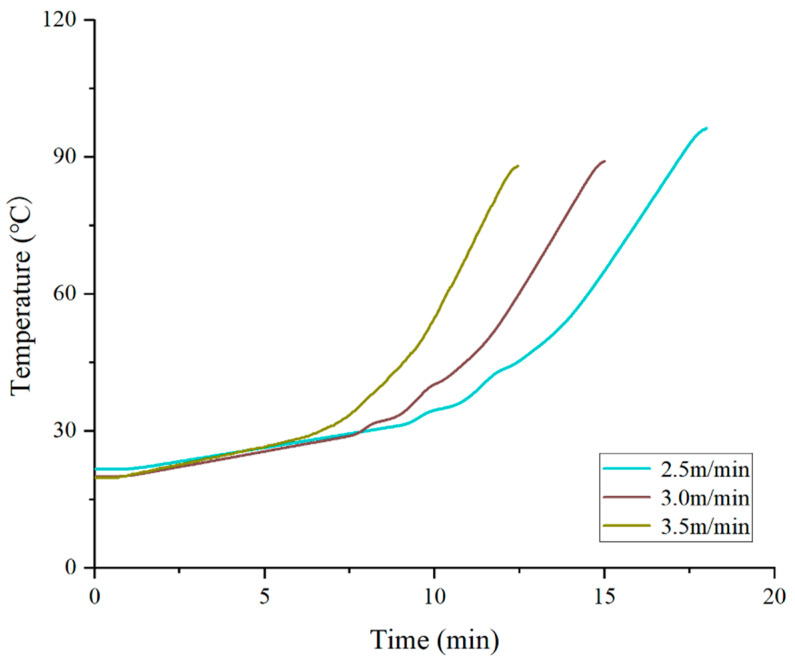
Effect of vehicle speed on the deep-layer temperature of pavement.

The effect of vehicle speed on the temperature at a depth of 4 cm is also significant, but the pattern differs from that of the pavement surface temperature. As shown in Figure 9, the temperatures at a 4 cm depth after 15 min of heating corresponding to speeds of 2.5 m/min, 3.0 m/min, and 3.5 m/min are 96.2 °C, 89.1 °C, and 87.7 °C, respectively. When the vehicle speed is reduced from 3.0 m/min to 2.5 m/min, the temperature at a 4 cm depth increases by 7.1 °C, whereas the pavement surface temperature increases by only 4.5 °C—a trend that is exactly opposite to the behavior of the previous two parameters. Under low-speed conditions, the marginal benefit for deep-layer heating is actually higher because the longer heating time allows heat to be fully conducted downward rather than merely accumulating in the surface layer [23].

This finding has important implications for intermittent heating processes: if an increase in deep-layer temperature is required, reducing the vehicle speed is more effective than raising the hot air temperature. For example, reducing the vehicle speed from 3.0 m/min to 2.5 m/min increases the temperature at the 4 cm depth by 7.1 °C, while the surface temperature increases by only 4.5 °C. In contrast, increasing the hot air temperature from 550 °C to 600 °C raises the temperature at the 4 cm depth by only 3.7 °C, but the surface temperature increases by 4.2 °C. Therefore, when deep-layer temperature is insufficient, priority should be given to appropriately reducing the vehicle speed (while ensuring that the peak surface temperature does not exceed the limit), followed by increasing the hot air temperature.

### 3.3. Sensitivity Analysis of Heating Parameters

This section uses a data-driven approach to quantify the relationships between process parameters and temperature responses, and then provides optimization recommendations for engineering applications. Taking the pavement surface temperature (*T_s_*) and the temperature at a depth of 4 cm (*T_d_*) after 15 min of heating as dependent variables, and the hot air temperature (*T*_0_, °C), hot air velocity (*v_a_*, m/s), and heating vehicle speed (*v_m_*, m/min) as independent variables, a linear regression analysis was conducted. The regression equations are as follows:

*T_s_* = 117.56 + 0.097*T*_0_ + 3.95*v_a_* − 8.50*v_m_* (R^2^ = 0.998)

*T_d_* = 20.38 + 0.115*T*_0_ + 2.80*v_a_* − 8.50*v_m_* (R^2^ = 0.997)

The regression model exhibits an excellent goodness of fit for predicting both the pavement surface temperature and the temperature at a depth of 4 cm, with coefficients of determination (R^2^) of 0.998 and 0.997, respectively. This indicates that the linear model accurately captures the relationships between the three heating parameters and the temperature responses, with small residuals. From the regression coefficients, for every 1.0 °C increase in hot air temperature, the pavement surface temperature increases by an average of 0.097 °C, while the temperature at a 4 cm depth increases by 0.115 °C. This suggests that the deep layer is slightly more sensitive to hot air temperature than the surface, which is consistent with the physical mechanism of downward heat conduction. For every 1.0 m/s increase in hot air velocity, the surface temperature rises by an average of 3.95 °C, and the deep-layer temperature rises by an average of 2.8 °C, reflecting the stronger direct effect of an increased convective heat transfer coefficient on surface heating. For every 1 m/min decrease in the heating plate travel speed, both the surface temperature and the deep-layer temperature increase by an average of 8.5 °C, indicating that vehicle speed has an identical quantitative effect on the temperature rise at both depths. This is attributed to the equivalent increase in heat input resulting from the extended heating time under intermittent heating mode. Therefore, this regression equation can be used not only for parameter sensitivity ranking but also as a rapid prediction tool, providing a quantitative basis for optimizing parameter combinations in hot in-place recycling construction.

### 3.4. Thermal Penetration Efficiency

The thermal penetration efficiency is defined as η = *T_d_*/*T_s_*, where *T_d_* and *T_s_* are the temperature rises at a 4 cm depth and the pavement surface, respectively [24]. The calculated values of η for each working condition are shown in Table 2. It can be seen that the most effective way to improve the heat penetration efficiency is to reduce the speed of the heating machine or increase the hot air temperature. However, the speed of the hot air has little effect on the improvement of penetration efficiency. To prevent the surface temperature from getting too high, it is recommended to further reduce the moving speed of the heating equipment.

### 3.5. Comprehensive Suggestions for the Intermittent Heating Process

Based on the above analysis, the following recommendations are proposed for the intermittent heating process in asphalt pavement hot in-place recycling:

(1) Parameter prioritization strategy

Based on parameter sensitivity and thermal penetration efficiency, when heating is insufficient, priority should be given to raising the hot air temperature and lowering the vehicle speed. If the aim is to further enhance the deep-layer temperature, it is recommended to reduce the vehicle speed first rather than continuously raising the hot air temperature, in order to prevent the occurrence of surface carbonization.

(2) Sawtooth pattern control for intermittent heating

The peak temperature of the sawtooth pattern is recommended to be controlled within 190–200 °C, and the trough temperature should not fall below 150 °C. A trough temperature that is too low indicates excessive heat loss; in such cases, the intermittent cycle should be shortened or ambient thermal insulation measures should be enhanced.

## 4. Conclusions

Based on the above analysis, the main conclusions of this study are as follows:

- Travel speed has the largest absolute regression coefficient of 8.5 °C·min/m, making it the most sensitive parameter for temperature regulation, followed by hot air temperature, while hot air velocity has the smallest effect. This means that adjusting the vehicle speed is the most convenient way to control the surface and deep-layer temperatures of pavement during hot in-place recycling.
- The ratio of deep-layer temperature rise to surface temperature rise varies with the parameter. When the hot air temperature is increased, the ratio ranges from approximately 1.2 to 1.4, indicating a greater benefit for the deep layer. When hot air velocity is increased, the ratio ranges from 0.6 to 0.7, meaning that heat mainly remains in the surface layer. When vehicle speed is reduced, the ratio is approximately 1.0, indicating an equal increase at both depths.
- The thermal penetration efficiency increases with increasing outlet air temperature, increasing hot air velocity, and decreasing vehicle speed. However, as the hot air temperature continues to rise, the benefits of the deep-layer temperature gradually decrease and there is a risk of surface carbonization. While the marginal benefit for deep-layer heating is actually higher under low-speed conditions.
- In the next step, field engineering will be employed to validate the accuracy of the regression equation established in this study in predicting the pavement surface and deep-layer temperatures. Based on the validation results, the regression equation will be further calibrated to develop precise guidance regarding heating parameters for hot in-place recycling.

## Figures and Tables

**Table 1:** Selection of different heating parameter values.

Parameters	Values		
Hot air temperature (°C)	500	550	600
Hot air velocity (m/s)	12	13	14
Heating vehicle speed (m/min)	2.5	3.0	3.5

**Table 2:** Thermal penetration efficiency under different working conditions.

Parameter Variation	The Trend of η
*T*_0_ increases (500, 550, 600)	0.46→0.49→0.50
*v_a_* increases (12, 13, 14)	0.46→0.46→0.47
*v_m_* increases (2.5, 3.0, 3.5)	0.49→0.46→0.47

## Data Availability

The original contributions presented in this study are included in the article. Further inquiries can be directed to the corresponding author.
